# Oxytocin Enhances Time-Dependent Responses in the Aggressive Zebrafish (*Danio rerio*)

**DOI:** 10.3390/brainsci14030203

**Published:** 2024-02-22

**Authors:** Madalina-Andreea Robea, Georgiana Oprea, Gabriel Plavan, Mircea Nicusor Nicoara, Ioannis Mavroudis, Vasile Burlui, Alin Ciobica

**Affiliations:** 1 Doctoral School of Biology, Faculty of Biology, “Alexandru Ioan Cuza” University of Iasi, Bd. Carol I 20A, 700505 Iasi, Romania; madalina.robea11@gmail.com; 2 Department of Biology, Faculty of Biology, “Alexandru Ioan Cuza” University of Iasi, Bd. Carol I 20A, 700505 Iasi, Romania; opreageorgiana801@yahoo.ro (G.O.); gabriel.plavan@uaic.ro (G.P.); mirmag@uaic.ro (M.N.N.); 3 Doctoral School of Geosciences, Faculty of Geography and Geology, “Alexandru Ioan Cuza” University of Iasi, Bd. Carol I 20A, 700505 Iasi, Romania; 4 Department of Neurology, Leeds Teaching Hospitals, NHS Trust, Leeds LS2 9JT, UK; i.mavroudis@nhs.net; 5 Faculty of Medicine, Leeds University, Leeds LS2 9JT, UK; 6 Preclinical Department, Apollonia University, Pacurari Street 11, 700511 Iasi, Romania; vburlui@gmail.com; 7 Academy of Romanian Scientists, 3 Ilfov, 050044, Bucharest, Romania; 8 Center of Biomedical Research, Romanian Academy, Iasi Branch, Teodor Codrescu 2, 700481 Iasi, Romania

**Keywords:** oxytocin, autism spectrum disorder, aggressive behavior

## Abstract

Autism spectrum disorder (ASD) has become one of the most well-known disorders encountered since early childhood among people. Nowadays, the main concerns are its high prevalence and the lack of proper therapeutic interventions. In this way, the necessity of using animal models that can mimic some of the spectrum symptoms, besides deepening the mechanisms of occurrence, is undeniable. Oxytocin (OT) is often mentioned and linked to producing social domain improvements. The goal of the present study was to determine if different time exposures to OT can trigger distinct behavioral responses in zebrafish, potentially offering insights into autism therapy. To accomplish this goal, zebrafish were exposed to the same dose of OT (33.2 ng/mL OT) for one week but with different time frames, such as: continuous exposure for seven days, fifteen minutes per day for seven days, and every two days for the same amount of time. The behavior of the fish was recorded using the EthoVision XT 11.5 software, and each trial lasted four minutes. Specific parameters for locomotor activity and aggressive behavior were measured. Overall, zebrafish exposure to OT generated several improvements in locomotor activity and aggressive behavior. Moreover, the differences in the exposure period indicated that time is an important factor, showing that continuous exposure to OT was linked with better performance than exposure to the hormone every two days. At the same time, the most variable results were observed in the case of fish exposed every day to OT. Exposure to OT could lead to certain improvements in zebrafish behavior that can be time-sensitive. Nevertheless, further work is needed in order to investigate the mechanisms of action of OT in an ASD context.

## 1. Introduction

Today, autism spectrum disorder (ASD), which is defined as a complex neurodevelopmental condition, is estimated to be closer to 2% of the world population regarding the rate of diagnosis [1]. Moreover, 1 in 44 children is diagnosed based on three levels of severity specific to ASD classification, with a higher prevalence among boys (a sex ratio of one girl per four boys) [1,2,3,4]. According to the last edition of the Diagnostic and Statistical Manual of Mental Disorders (DSM-V, 2013), there are two criteria that are evaluated in order to diagnose ASD: persistent deficits in social communication and social interaction and restricted, repetitive patterns of behavior, interests, or activities [2]. Even if it is well known that ASD is mainly caused by genetic mutations, in recent years, the environmental implications have been highlighted [5,6,7]. Due to its multifactorial character and its complexity, ASD requires a high level of attention from authorities, citizens, and medical specialists. Furthermore, research in this field of ASD was made using animal models that were capable of developing and sustaining certain autistic traits usually seen in humans [8,9,10,11]. For instance, zebrafish (*Danio rerio*) is a popular animal model that has been used almost in every domain of medical research due to its suitability [12,13,14]. Since its first use in the 1970s by George Streisinger, zebrafish has contributed to the present knowledge, mostly because of the advantages it possesses such as a transparent embryo stage, external fertilization, rapid development, high fecundity, easy genetic manipulation, and a fully sequenced genome, among others [15,16]. There are numerous reports that describe the involvement of zebrafish in ASD modeling for mechanisms and therapeutic intervention studies [10,17]. Zebrafish have a high repertoire of behaviors that are extensively studied, especially those that are strongly connected to the disorder. In ASD cases, sociability is the main criteria for developing an adequate animal model. In this way, zebrafish, known for its sociable character, presents varied social behaviors (shoaling and schooling) that are exhibited in its group or when it is involved in a new group formation [18,19,20]. For the present study, it was chosen to analyze the level of aggression that could be displayed during or after exposure. This type of behavior has been studied in zebrafish through numerous experimental tests [21,22,23]. Furthermore, aggression is often reported in ASD cases by parents and medical specialists, and consequently, this behavior has a negative impact on the quality of life of people with autism [24]. It was also outlined that boys are more predisposed to manifest a certain form of aggression [3,25].

Despite the fact that ASD does not yet have a cure, there are different strategies to ameliorate the symptoms of autism, including pharmacological and nonpharmacological ones. The lack of treatment has made it almost impossible to improve these symptoms, which can be achieved by taking adjustable amounts of antipsychotics, antidepressants, or other types of drugs. For example, the use of risperidone and aripiprazole (dopaminergic agonists) proved effective in treating impulsivity and aggression [17]. In addition, for the social symptoms of ASD, the use of oxytocin (OT) was linked to being a good intervention. Produced in the hypothalamus and released by the pituitary gland in the bloodstream, OT is a neuropeptide hormone that is implicated in social cognition, social behaviors, and fear conditioning [26,27,28]. Synthetized primarily in the neurons of hypothalamus, OT has receptors all over the brain [26]. It was proved that administration of OT can lead to significant improvements in memory of people diagnosed with Asperger syndrome (which is an old type of ASD classification) by helping them to reduce repetitive behaviors, remember human faces, distinguish emotions, or even make speech intonation modifications [29,30]. Studies using the “Childhood Autism Rating Scale” found that plasma OT levels are lower in children with ASD compared to those with typical developmental processes. In a physiological condition, OT actions are reflected in bond formation by facilitating it or in the process of making new social connections that lead to a more rewarding feeling [31]. In addition, OT is linked to the childbirth process by stimulating uterine contractions and lactation after childbirth [32]. While OT increases in a physiological state, it was demonstrated that this hormone presents lower levels in the mothers of neurotypical children [33]. Although OT can be used to relieve ASD symptoms, it is not administered as a therapeutic tool. The lack of research trials and the questionable data that was already published highlight the necessity of further investigations for its positive potential. According to a recent review, the level of OT in blood serum tends to be lower in children with autism [34]. Approaches for using OT on people with ASD started to be explored in multiple clinical studies. Intranasal administration of OT was chosen to be the easiest and the most efficient way due to the capacity of neuropeptides to cross the blood–brain barrier and, consequently, facilitate the path to the brain structures [35,36,37]. The administration of 0.4 IU (International Units)/kg/dose for three months contributed to significant improvements in the behavior of children with ASD and adolescents [38]. Similar data were published after six weeks of treatment with 24 IU that was associated with behavioral therapies [39]. The most recent study offered by Guastella et al. that explored the efficacy, tolerability, and safety of intranasally-administered OT reported certain effects in young children with autism [40]. Although OT is frequently linked with social domain, it can influence other brain processes such as memory, fear, adaptive behavior, or anxiety. A recent study from 2023 concluded that a single dose of OT (24 IU) can manipulate the acquisition of intrusive memories in healthy young females exposed to an analogue trauma, but not the consolidation of these memories [41]. The amygdala, the center of emotions, involved in the acquisition, storage, expression and extinction of fear memories is also modulated by OT [42,43]. It was proved that OT is capable of reducing amygdala activity by increasing social interactions and reducing anxiety and fear levels in humans [44,45]. In rodent models, OT has been associated with improvements in long-lasting spatial memory, hippocampal synaptic plasticity, and contextual fear memory after administering different hormone doses [46,47,48].

Despite mentioning the beneficial impact of OT, there are several reported issues regarding the standardization or even in respect to the validation of OT measurements through different methodologies. For instance, measuring the OT levels in human plasma, saliva, urine, or in cerebrospinal liquid requires a lot of attention from specialists, taking into account the diversity of assay techniques for quantifying OT [49]. Moreover, according to Quintana et al., the small sample sizes, the absence of theories that elaborate on more than OT’s role in social domain, and the inconsistent use of pre-registration are some of the factors that contributes to the lack of OT reproducibility in experimental research [50]. Besides this, it is important to consider other aspects of OT measurement in peripheral fluids, such as biological clearance or temporal resolution, performing a correlation with OT brain concentrations, the potential of OT to bind to other matrix components, and the possibility of interferants during the process of measurement [51]. Given these particular “criteria”, animal models have been used to study the effects of different substances on the OT receptors in humans. Both rodents and fish were involved in replicating OT-influenced social behaviors within ASD symptomology because of the neuropeptide existence. Long conserved across evolution, OT has many replacements: isotocin in fish or mesotocin in reptiles and birds [52]. In zebrafish, the orthologue of OT is linked to increased social behavior and reduced fear of predators [53,54]. According to Gemmer et al. [55], OT participates in the process of development and maintenance of zebrafish sociability. This fact was proven when the two OT receptors (Oxtr and Oxtrl) knocked-out in fish exhibited impaired social behavior. Moreover, a protective effect of OT was observed when zebrafish embryos were exposed to valproic acid, given as an inductor for ASD. An amount of 50 µM OT was the most appropriate concentration that determined this effect, which was correlated with increased expression of shank3a, shank3b, and OT receptor genes [56].

On the other hand, aggression, as antisocial behavior, was mentioned multiple times in ASD cases as a way to express emotions [24,57,58]. For instance, enhanced aggression forms against an intruder is a common pattern of maternal behavior that is specific to all females from the animal kingdom [59,60,61]. In 2015, Bosch et al. concluded that the release of OT is more critical for maternal behavior regulation than the differences observed in brain OT receptors in a rat model [62]. Similar results were reported in 2014 by Sabihi et al. after a maternal defense test, which explored the brain areas involved in female rats aggression displayed in postpartum days [63]. Evidence showed that OT is capable of adjusting zebrafish aggression in different stress conditions by the presence of OT axons in various brain areas linked to anxiety and aggression [64].

Therefore, the present study intended to investigate the effect of OT on zebrafish locomotor activity and aggressive behavior. Continuous exposure for fifteen minutes every day and fifteen minutes every two days at the same dose (33.2 ng/mL) was selected to evaluate the OT impact on zebrafish.

## 2. Materials and Methods

### 2.1. Animals

Animals were purchased and accommodated in the facility for a period of three weeks. For this study, a total of 150 zebrafish (*Danio rerio*, WT AB, 6–7 months, sex ratio 1:1) were used. Fish were subjected to 14:10 h light–dark at a constant temperature of 26 ± 0.5 °C. The water from housing and experimental tanks was changed daily to avoid the accumulation of organic products, along with the quality parameters (a pH of 7.5, a dissolved oxygen index of 7.20 mg/L, conductivity of 551 µS/cm, and ammonia of 0.05 mg/L) [65,66]. Fish were fed twice a day with the special formula of TetraMin flakes. This study followed the requirements set by the Commission of the European Union and the Directive of the European Parliament of the 22 September 2010 Council [67,68]. Consequently, this study was approved by the Ethics Commission of the Faculty of Biology with full No. 1272/19.05.2023.

### 2.2. Chemicals

Oxytocin was purchased in liquid form from the Pasteur company (Filipesti, Romania). The dose of OT was determined by studying the existing literature and set at 33.2 ng/mL [56,69]. The compound was directly dissolved in the medium by adding a certain amount of OT to the system water. The water was changed daily, and OT was added where that was the case.

### 2.3. Behavioral Test Procedure

**Figure 1:**
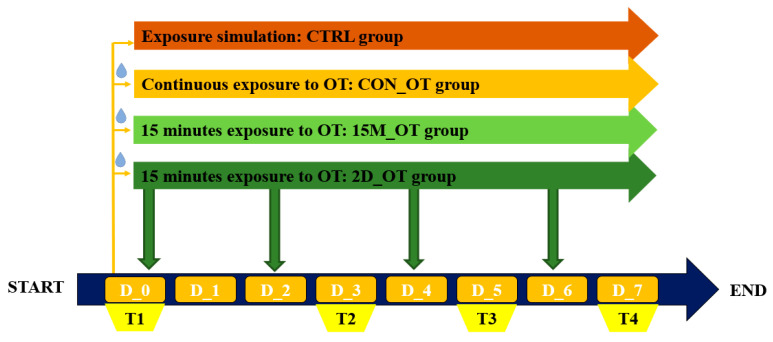
The exposure protocol to oxytocin and testing diagram. Fish were exposed to three different time frames for oxytocin: continuous (CON_OT group), fifteen minutes every day (15M_OT group), and fifteen minutes every two days (2D_OT group). Also, a control group was added (the CTRL group). The behavioral testing was scheduled for D_0, known to be the initial behavioral evaluation, and the third, fifth, and seventh days for compound effect quantification. D stands for day and T for testing.

Before exposure to OT, zebrafish were randomly divided and acclimated in experimental tanks for 1 week. Then, four groups were established: control and three groups with different time exposure to OT as continuous (CON_OT), fifteen minutes (15M_OT), and every two days (2D_OT) exposure for an experimental period of seven days (Figure 1). The control group was established to assure that all the behavioral changes were caused by OT exposure and no other factors (environmental factors, for instance). Also, this group simulated OT exposure every day by changing the medium in order to be replaced with a fresh one. The same method was applied for the group with continuous exposure to OT. Each group had 10 animals. Except for the fish with continuous exposure, the other two groups were transferred to another tank and exposed in a similar manner to the previous group to OT for fifteen minutes every day or every two days. To record the initial behavior before exposure to OT, locomotor activity and aggression tests were conducted. After this phase, fish were exposed to OT according to the protocol and tested on the third, fifth, and last day of the experiment. At the end of the study, the fish were sacrificed by immersion in iced water, according to the procedures of the European Union [67].

#### 2.3.1. The Locomotor Activity Test

**Figure 2:**
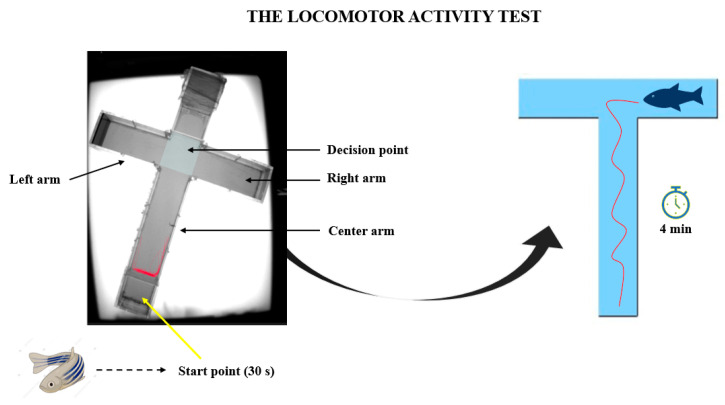
The representation of locomotor activity test performed during the study to assess the changes that occurred after oxytocin exposure.

The locomotor activity test was assayed using the cross maze provided by Noldus Company that accompanied the EthoVision XT 11.5 software (Wageningen, Netherlands). The cross maze was transformed into a T-maze and divided into three arms as left, right, and center. A start point was established in the center arm, while the decision point was selected to be at the arms intersection (Figure 2). This test was performed in order to observe the changes that occurred in the zebrafish locomotion parameters. For this aim, several parameters were chosen, such as the total distance traveled, average swimming velocity, time spent inactive, clockwise and counter clockwise rotations, angular velocity, meander, and turn angle. Each trial consisted of a four minute recording that was further analyzed in real time through the behavioral software.

#### 2.3.2. The Aggression Test

**Figure 3:**
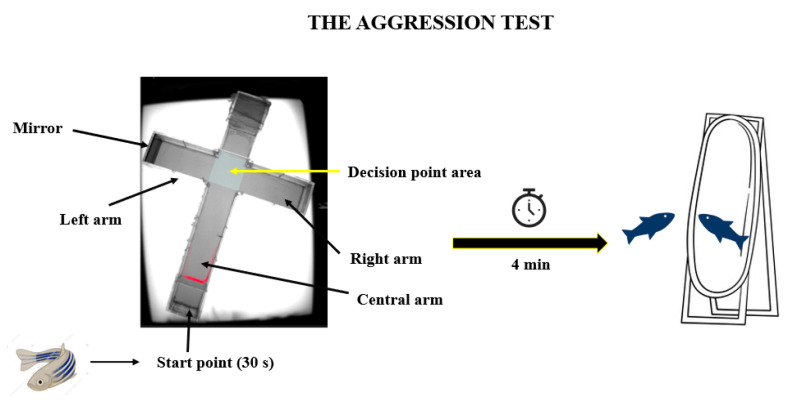
The representation of aggression test performed during the study to evaluate the experimental groups behavior.

To analyze the intensity of the aggression behavior of the zebrafish treated with OT, an aggression test was performed. For this test, the same experimental maze was used but adapted in order to measure the targeted behavior. To trigger the specific behavior, a mirror was added to the left arm as stimulus (Figure 3). The existing literature often mentions the use of a mirror as a tool to observe the zebrafish’s aggression, due to the fact that the fish is not able to recognize its reflection in the mirror and therefore treats it as a possible opponent [22,23]. The time spent by the fish in the arms of the maze was measured, especially the time spent in the left arm in the area dedicated to the mirror. This behavior was recorded for a period of four minutes using the EthoVision XT 11.5 software.

### 2.4. Quantification of Behavior and Statistical Analysis

All the behavioral data were analyzed by the Shapiro–Wilk test to check the normality of data distribution. When this assumption was satisfied, a one-way analysis of variance (ANOVA) followed by the Tukey’s test evaluated the differences recorded between the variables. The *p* value was set at 0.05, and all the values below it indicated statistical significance. All results were presented as mean ± standard error of the mean (SEM). Data processing and sorting have been achieved through the Excel spreadsheets from the Microsoft Office Professional Plus 2018 package (Microsoft Corporation, Redmond, WA, USA). The statistical processing and graphical representation of the results were carried out using the OriginPro 11.5 software, 2021 (OriginLab Corporation, Northampton, MA, USA).

## 3. Results

### 3.1. Oxytocin Has Time-Dependent Effects on Zebrafish Locomotion

**Figure 4:**
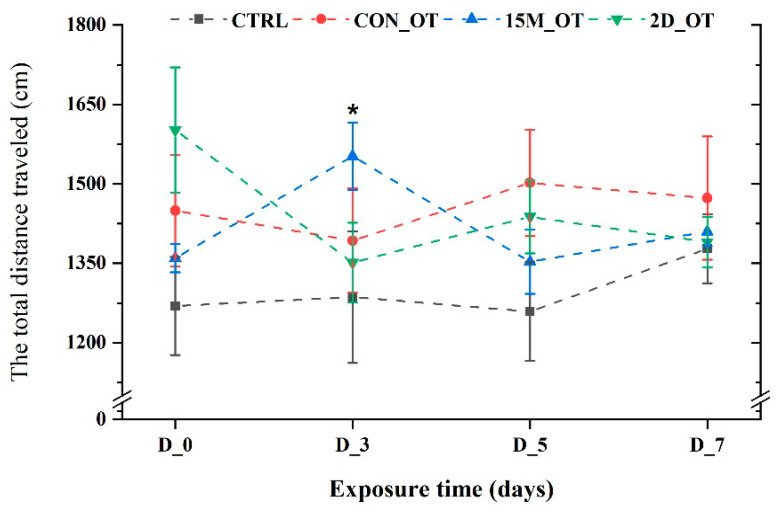
The total distance traveled by the experimental groups during the locomotor activity test. D stands for day and OT for oxytocin, gray line: control, red line: continuous exposure to OT (CON_OT), blue line: fifteen min exposure every day (15M_OT), green line: fifteen min exposure every two days (2D_OT). The data are expressed as mean ± SEM (n = 10); * *p* < 0.05 ANOVA, Tukey is significant compared to the results from D_0 that were set as the initial behavior of each experimental group.

**Figure 5:**
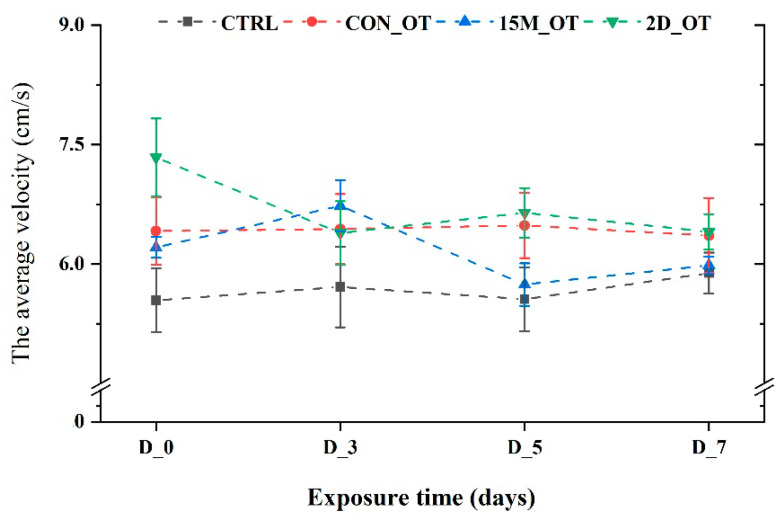
The average velocity of the experimental groups during the locomotor activity test. D stands for day and OT for oxytocin, gray line: control (CTRL), red line: continuous exposure to OT (CON_OT), blue line: fifteen min exposure every day (15M_OT), green line: fifteen min exposure every two days (2D_OT). The data are expressed as mean ± SEM (n = 10); *p* > 0.05 ANOVA and compared to the results from D_0 that were set as the initial behavior of each experimental group.

The first parameter measured for locomotor activity was the total distance traveled by the fish during the trials. There were no significant changes in this parameter values for CTRL and the group exposed continuously to OT (*p* = 0.31 and *p* = 0.19 ANOVA, Tukey’s test). In contrast, the group exposed to 15 min every day showed an increase in the distance traveled in the third day compared to the initial behavior (1552.2 ± 63.1 cm vs. 1359.7 ± 26.7 cm, *p* = 0.03 ANOVA, Tukey’s test) (Figure 4). While the fish exposed every day for fifteen min to OT returned to similar activity as that seen in the pretreatment stage, the fish that received the hormone every two days exhibited a decrease in the distance traveled: D_3 (1351.1 ± 75.1 cm), D_5 (1437.8 ± 69.2 cm), and D_7 (1390.1 ± 47.7 cm), compared to the initial data: 1601.8 ± 118.1 cm (*p* > 0.05 ANOVA). No significant changes were noted for the average velocity parameter of the zebrafish groups (*p* > 0.05 ANOVA) (Figure 5).

**Figure 6:**
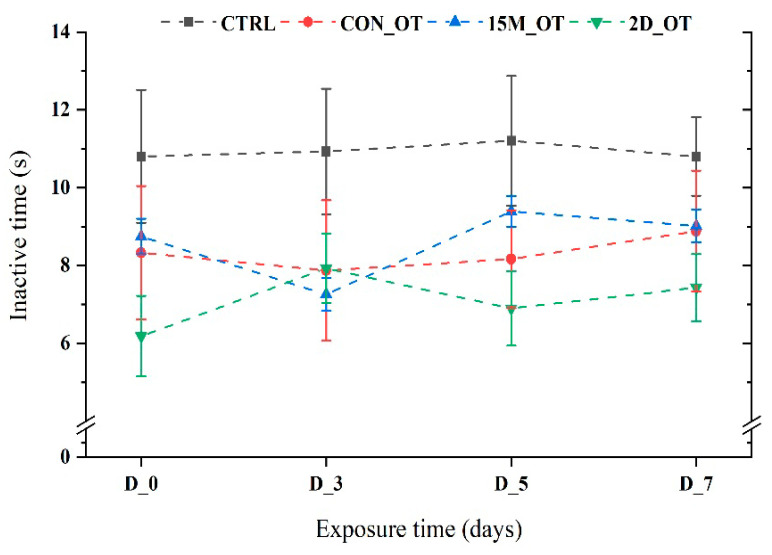
The inactive time of the experimental groups during the locomotor activity test. D stands for day and OT for oxytocin, gray line: control (CTRL), red line: continuous exposure to OT (CON_OT), blue line: fifteen min exposure every day (15M_OT), green line: fifteen min exposure every two days (2D_OT). The data are expressed as mean ± SEM (n = 10); *p* > 0.05 ANOVA and compared to the results from D_0 that were set as the initial behavior of each experimental group.

In regard to the time spent moving, the fish did not present any important modifications after exposure to OT (*p* > 0.05 ANOVA). As can be seen from Figure 6, the most inactive group was the control that simulated exposure to OT, while the OT groups exhibited different trends. For instance, 15 min of daily exposure led to a short time decrease in the inactivity time (7.2 ± 0.4 s, *p* = 0.07 ANOVA, Tukey’s test) when comparing the initial behavior (8.7 ± 0.4 s) with the other two testing days: D_5: 9.3 ± 0.3 s (*p* = 0.005 ANOVA, Tukey’s test) and D_7: 9 ± 0.4 s (*p* = 0.02 ANOVA, Tukey’s test). On the other hand, the last group that received OT every two days did not display any significant effect of the compound, being less inactive for the majority of time except for D_3 (7.9 ± 0.8 s, *p* = 0.56 ANOVA, Tukey’s test) vs. initial behavior (6.1 ± 1.03 s) (Figure 6).

**Figure 7:**
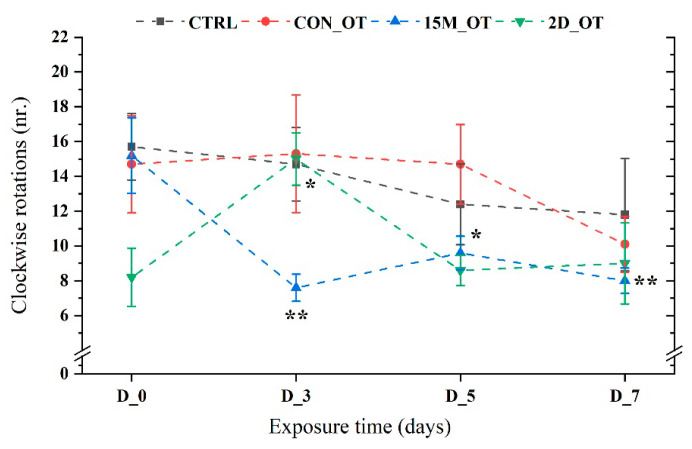
The number of clockwise rotations made by the experimental groups during the locomotor activity test. D stands for day and OT for oxytocin, gray line: control (CTRL), red line: continuous exposure to OT (CON_OT), blue line: fifteen min exposure every day (15M_OT), green line: fifteen min exposure every two days (2D_OT). The data are expressed as mean ± SEM (n = 10); * *p* < 0.05, ** *p* < 0.01 ANOVA, Tukey is significant compared to the results from D_0 that were set as the initial behavior of each experimental group.

Clockwise rotations (right-hand) were generally similar to the initial behavior for the control fish and for the group exposed continuously to OT (*p* = 0.57, respectively, *p* = 0.86 ANOVA). Although no important effects were observed for the CON_OT group, 15M_OT exhibited a lower number of rotations in all the experimental days (D_3: 7.6 ± 0.7, *p* = 0.001 ANOVA, Tukey’s test; D_5: 9.6 ± 0.9, *p* = 0.02 ANOVA, Tukey’s test; D_7: 8 ± 0.7 *p* = 0.002 ANOVA, Tukey’s test), when compared to the initial behavior (15.2 ± 2.1) (Figure 7). Regarding the last group rotations, this parameter recorded a significant value for D_3 (15 ± 1.5, *p* = 0.03 ANOVA, Tukey’s test) vs. CTRL (8.2 ± 1.6), with no more changes until the end of the study.

**Figure 8:**
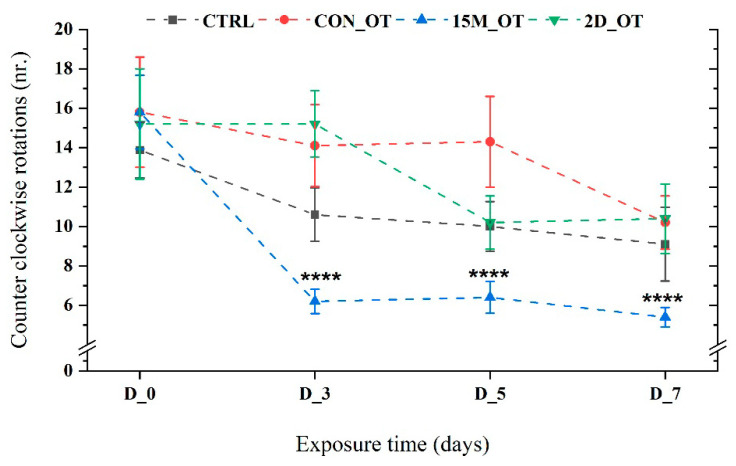
The number of counter clockwise rotations made by the experimental groups during the locomotor activity test. D stands for day and OT for oxytocin, gray line: control (CTRL), red line: continuous exposure to OT (CON_OT), blue line: fifteen min exposure every day (15M_OT), green line: fifteen min exposure every two days (2D_OT_). The data are expressed as mean ± SEM (n = 10); **** *p* < 0.001 ANOVA, Tukey is significant compared to the results from D_0 that were set as the initial behavior of each experimental group.

When the counter clockwise rotations (left-hand) were measured, the CTRL, CON_OT, and 2D_OT presented a similar activity to the right-hand rotations (*p* = 0.98, *p* = 0.32, respectively, *p* = 0.45 ANOVA). The number of rotations for the 15M_OT group decreased significantly when compared to the initial behavior (15.8 ± 1.8) vs. D_3: 6.2 ± 0.6, *p* = 0.0001 ANOVA, Tukey’s test; D_5: 6.4 ± 0.8, *p* = 0.0001 ANOVA, Tukey’s test; D_7: 5.4 ± 0.49, *p* = 0.0001 ANOVA, Tukey’s test) (Figure 8).

**Figure 9:**
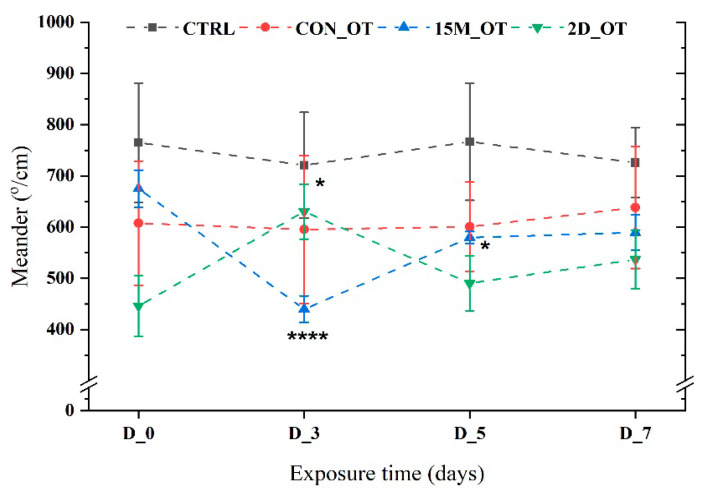
The meander parameter of the experimental groups during the locomotor activity test. D stands for day and OT for oxytocin, gray line: control (CTRL), red line: continuous exposure to OT (CON_OT), blue line: fifteen min exposure every day (15M_OT), green line: fifteen min exposure every two days (2D_OT). The data are expressed as mean ± SEM (n = 10); * *p* < 0.05 and **** *p* < 0.01 ANOVA, Tukey is significant compared to the results from D_0 that were set as the initial behavior of each experimental group.

The meander parameter, an indicator of swimming pattern, did not reveal important OT effects on zebrafish. As is shown in Figure 9, the CTRL and CON_OT groups had an almost linear trend with no observable activity alternations (*p* = 0.23 and *p* = 0.47 ANOVA). Significantly more changes occurred for the 15M_OT fish that express a decrease in D_3 (439.5 ± 25.9°/cm, *p* = 0.0001 ANOVA, Tukey’s test), followed by D_5 (579.5 ± 11.7°/cm, *p* = 0.02 ANOVA, Tukey’s test) in comparison to D_0 (675.1 ± 35.9°/cm) (Figure 9). An opposite activity was presented by the 2D_OT fish that recorded a high peak in D_3 (630.2 ± 53.5°/cm, *p* = 0.03 ANOVA, Tukey’s test) vs. D_0 (445.9 ± 59.6°/cm). In the end, the fish returned to their normal state, as shown by the natural behavior in D_0 (Figure 9).

**Figure 10:**
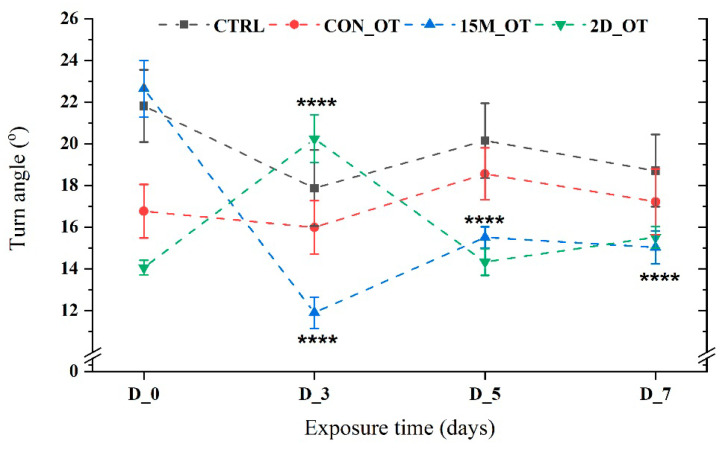
The turn angle parameter of the experimental groups during the locomotor activity test. D stands for day and OT for oxytocin, gray line: control (CTRL), red line: continuous exposure to OT (CON_OT), blue line: fifteen min exposure every day (15M_OT), green line: fifteen min exposure every two days (2D_OT). The data are expressed as mean ± SEM (n = 10); **** *p* < 0.01 ANOVA, Tukey is significant compared to the results from D_0 that were set as the initial behavior of each experimental group.

A significant effect triggered by the OT in the group exposed daily for 15 min was recorded for the turn angle parameter. Compared to the initial behavior (22.6 ± 1.35 °), the rate of turn decreased for all the experimental days as follows: D_3 (11.8 ± 0.7°, *p* = 0.0001 ANOVA, Tukey’s test), D_5 (15.5 ± 0.5°, *p* = 0.0001 ANOVA, Tukey’s test), and D_7 (15.02 ± 0.7°, *p* = 0.0001 ANOVA, Tukey’s test) (Figure 10). In contrast, the 2D_OT group exhibited an increase in turning rate on D_3 (20.2 ± 1.1°, *p* = 0.0001 ANOVA, Tukey’s test) compared to D_0 (14.1 ± 0.35°) and the activity on the other experimental days (Figure 10). There were no changes for the CTRL and CON_OT groups (*p* = 0.95 and *p* = 0.63 ANOVA).

**Figure 11:**
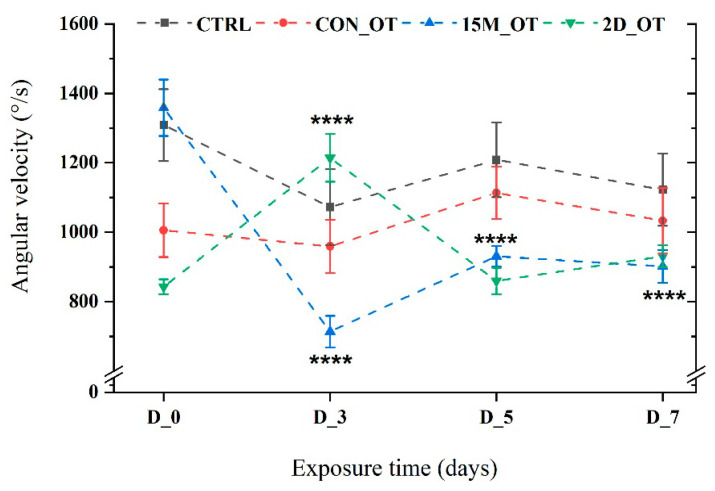
The angular velocity of the experimental groups during the locomotor activity test. D stands for day and OT for oxytocin, gray line: control (CTRL), red line: continuous exposure to OT (CON_OT), blue line: fifteen min exposure every day (15M_OT), green line: fifteen min exposure every two days (2D_OT). The data are expressed as mean ± SEM (n = 10); **** *p* < 0.01 ANOVA, Tukey is significant compared to the results from D_0 that were set as the initial behavior of each experimental group.

Finally, to complete the swimming pattern of the fish, angular velocity was also measured. Similar to the previous parameter, the activity of the fish exposed daily for 15 min indicated a reduction in the angular velocity values as compared to D_0 (1358.4 ± 81.3°/s) for D_3 (713.9 ± 45.1°/s, *p* = 0.0001 ANOVA, Tukey’s test), D_5 (930.8 ± 30.2°/s, *p* = 0.0001 ANOVA, Tukey’s test), and D_7 (901.7 ± 47.1°/s, *p* = 0.0001 ANOVA, Tukey’s test) (Figure 11). The group exposed to OT every two days had, for the majority of the time, similar activity to that of D_0 (843.2 ± 21.3°/s), except for the third day (1214 ± 68.7°/s, *p* = 0.0001 ANOVA, Tukey’s test), which recorded an important increase, as can be seen in Figure 11.

### 3.2. Oxytocin Triggers Aggressiveness in Zebrafish

**Figure 12:**
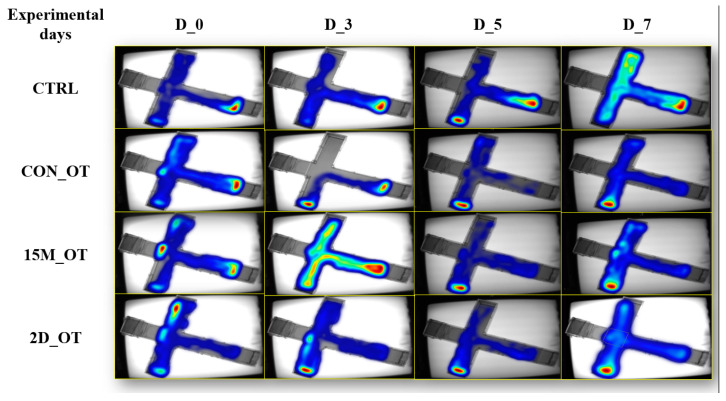
The time spent in the maze areas of the experimental groups, control (CTRL), continuous exposure to OT (CON_OT), fifteen min exposure every day (15M_OT), and fifteen min exposure every two days (2D_OT), during the aggression test by heatmap representations. D stands for day and OT for oxytocin. D_0 was considered as initial behavior.

In addition to the locomotor activity observations, the experimental groups were subjected to the aggression test, which evaluated the intensity of this behavior among the fish (Figure 12).

**Figure 13:**
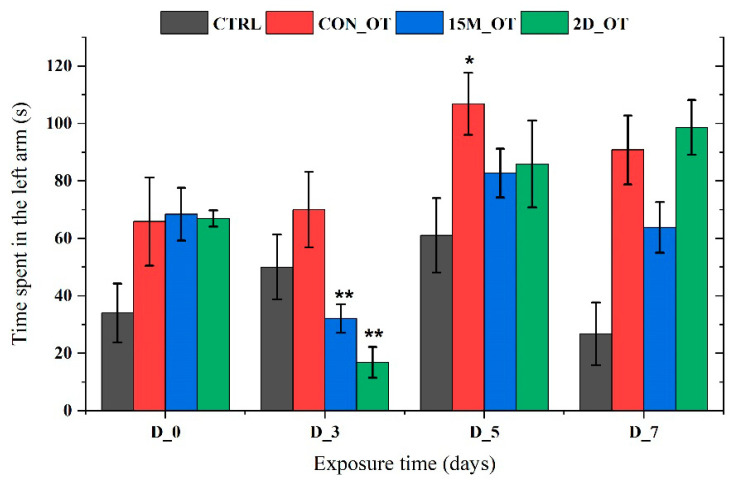
The time spent in the left arm by the experimental groups during the aggression test. D stands for day and OT for oxytocin, gray: control (CTRL), red: continuous exposure to OT (CON_OT), blue: fifteen min exposure every day (15M_OT), green: fifteen min exposure every two days (2D_OT). The data are expressed as mean ± SEM (n = 10); * *p* < 0.05, ** *p* < 0.01 ANOVA, Tukey is significant compared to the results from D_0 that were set as the initial behavior for each experimental group.

In line with this, the time spent in the left arm was quantified, and its graphical representation is shown in Figure 13. It seems that OT had decreased the time spent in the left arm of the 15M_OT (32.1 ± 4.9 s, *p* = 0.01 ANOVA, Tukey’s test) and 2D_OT (16.8 ± 5.3 s, *p* = 0.003 ANOVA, Tukey’s test) groups after the first three days of exposure compared to the natural behavior observed in D_0 (68.4 ± 9.1 s and 66.9 ± 2.8 s). In addition, the CON_OT group revealed an increase in the time spent next to the stimulus in D_5 (106.8 ± 10.7 s, *p* = 0.04 ANOVA, Tukey’s test) vs. D_0 (65.8 ± 15.3 s). There were no significant results for the CTRL group (*p* = 0.16 ANOVA).

**Figure 14:**
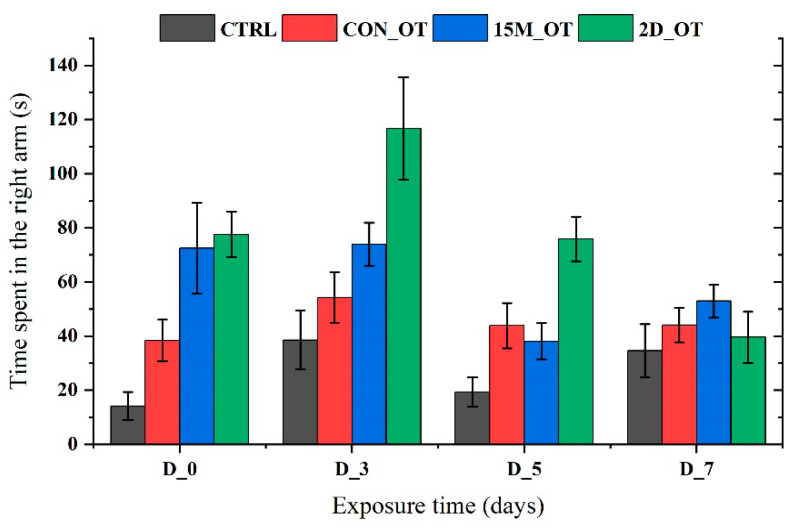
The time spent in the right arm by the experimental groups during the aggression test. D stands for day and OT for oxytocin, gray: control (CTRL), red: continuous exposure to OT (CON_OT), blue: fifteen min exposure every day (15M_OT), green: fifteen min exposure every two days (2D_OT). The data are expressed as mean ± SEM (n = 10) and compared to D_0 that were set as the initial behavior for each experimental group.

**Figure 15:**
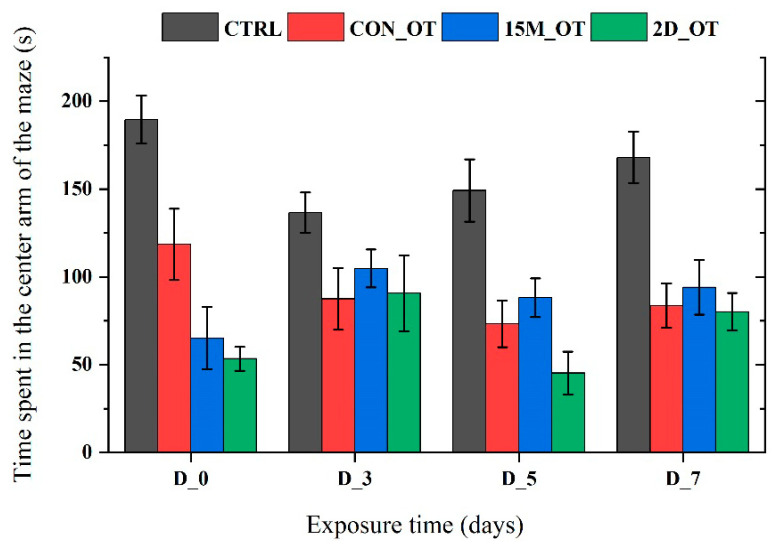
The time spent in the center arm by the experimental groups during the aggression test. D stands for day and OT for oxytocin, gray: control (CTRL), red: continuous exposure to OT (CON_OT), blue: fifteen min exposure every day (15M_OT), green: fifteen min exposure every two days (2D_OT). The data are expressed as mean ± SEM (n = 10) and compared to D_0 that were set as the initial behavior for each experimental group.

In addition to the time spent in the left arm, the time in the right, center, and decision point of the experimental groups were also measured. In Figure 14 and Figure 15, it can be seen that the time spent in the right and center arms of the maze by the fish did not reach significance according to ANOVA.

**Figure 16:**
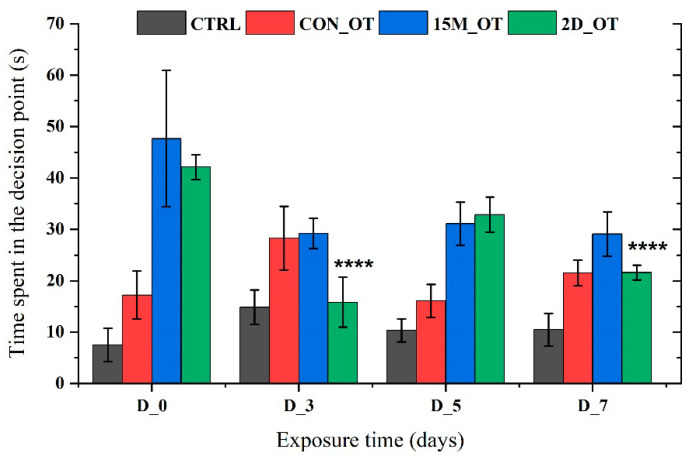
The time spent in the decision point by the experimental groups during the aggression test. D stands for day and OT for oxytocin, gray: control (CTRL), red: continuous exposure to OT (CON_OT), blue: fifteen min exposure every day (15M_OT), green: fifteen min exposure every two days (2D_OT). The data are expressed as mean ± SEM (n = 10); **** *p* < 0.01 ANOVA, Tukey is significant compared to the results from D_0 that were set as the initial behavior for each experimental group.

The time spent in the decision point arm did not reveal too many changes, except for the ones from the 2D_OT group. Consequently, the fish’s activity in this area had decreased in D_3 (15.8 ± 4.8 s, *p* = 0.0001 ANOVA, Tukey’s test) and D_7 (21.6 ± 1.4 s, *p* = 0.0001 ANOVA, Tukey’s test) vs. D_0 (42.1 ± 2.1 s), when the fish tended to spend more time before choosing their swimming direction (Figure 16).

## 4. Discussion

This work presents, for the first time, the effects triggered by different periods of exposure to OT in zebrafish. It was shown that continuous exposure to OT for seven days does not lead to significant changes in zebrafish locomotion and aggression levels. On the other hand, exposure to fifteen minutes per day for seven days exhibited increases for almost all the locomotor parameters studied, while exposure every two days pointed out opposite effects. Being a hypothalamic neuroendocrine peptide, OT actions have been described in numerous research studies, especially for its main implication in the social domain [26]. Many animal models were used in determining the mechanism of OT in the brain and its established connections with other brain cells and molecules. For instance, OT receptors can interact with the neurotransmitter system. When it is communicating with dopaminergic neurons, it favors the process of bond formation and interaction, which sparks the sensory and reward processes [56]. The OT controls the release of serotonin, another hormone engaged in stress control, anxiety, and social activity, while this hormone plays a similar role in encouraging OT release [70,71]. Known to be implicated more in the social domain, OT has been tested multiple times in zebrafish experiments. Exposure, administration, or evaluation of exogenous/endogenous OT indicated that it intervenes in the regulation of social and anxiety behavior in larval and adult zebrafish [54]. Recently, it was shown that OT receptors might be manipulated by age or by the social context of the fish, leading to different responses which cannot be pro- or antisocial [55].

This study aimed to evaluate if the duration of exposure can promote different responses in zebrafish activity. The first parameter studied for locomotor activity was the total distance traveled, which showed the most significant activity on the third day of the study for the group that received OT for fifteen minutes every day. The same cannot be said in the case of the fish exposed every two days to the hormone. While it recorded the highest distance in the pretreatment after just two doses of OT, the distance decreased considerably, a trend that was maintained until the end of the experimental period. Chuang et al. explored the role of OT in zebrafish by testing in three different environments, which were considered as stressful factors. The total distance swum during the novel tank diving test recorded a decrease when fish were staying in acidic water compared to double-ionized and high-ammonia environments. In addition, the tissues of zebrafish were evaluated through PCR, and a 38% increase and 58% decrease, respectively, in oxyrl expression were observed following the one-week treatment [64].

Regarding the time spent moving, the fish treated continuously and every day with OT tended to explore more of the maze compared to the activity observed in the initial behavior, while the last group recorded an increase in the time spent inactive. The exposure to L-368,899, an antagonist of OT receptors, proved that zebrafish locomotion was not affected, with no difference between the control and treated group. At the same time, the fish treated with the OT antagonist demonstrated a reduction in the time spent next to the conspecifics during social testing [54].

Another set of parameters that provided interesting results were the clockwise and counterclockwise rotations. In general, a higher number of rotations is associated with altered behavior by the repetitive or sequential character offered by the compound’s presence. In this study, fish exposed every day for fifteen minutes to OT revealed a reduction in the number of rotations, regardless of their type. Also, meander, turn angle, and angular velocity were investigated for describing the swimming pattern of the fish. These parameters express data about direction, the angle of fish head rotation, and the amplitude of movement. At the end of the study, OT-treated fish showed a decreased level of meandering, which suggests that hormone exposure had no a negative impact. A larger meander is linked with erratic movements, according to several studies where a neurotoxic impact was seen in zebrafish swimming patterns [72,73]. In humans, improvements in social and repetitive behaviors were observed after the administration of 24 IU OT once a day for four days and twice a day for six weeks [74,75]. When discussing OT involvement during the perinatal period, it was exhibited that alterations in OT could be reflected in suckling, child–mother bond establishment, or even in the first social reactions proposed as early signs for ASD [76]. The OT hormone can also act on the brain-derived neurotrophic factor (BDNF), according to some rodent studies, by increasing its expression in the hippocampus, which is further linked to brain plasticity that is essential for memory and learning [77,78]. Furthermore, Bukatova et al. observed that early exposure to OT can disturb the precursor and the mature forms of BDNF in male rat hippocampus [78].

Antisocial behaviors are often one of the common symptoms reported in ASD cases [24,58]. Genetically predisposed or environmentally induced aggression is associated with negative outcomes that, in general, perturb the lives of people with autism and those around them. There are findings that indicate that certain brain areas and connections are strongly correlated with the appearance of aggression. Reduced activity of the prefrontal cortex, lesions, or neuronal alterations were associated with violent aggression [79,80]. Malik et al. [81] described the involvement of specific OT receptor gene variants in the development or expression of this behavior and proved the tendency of males to manifest a higher risk for aggression. In line with this, Zhang et al. reported in 2018 on a Chinese male cohort that the OT receptor gene variant (rs237885) was significantly linked to increased risk of aggression and was sustained by childhood physical abuse as a result of its interaction [82]. In humans, the administration of OT offers controversial responses based on the existing literature. For example, acute administration of 24 IU OT to healthy male adults did not show any effect on aggressive behavior evaluated through “Point Subtraction Aggression Paradigm” at 30 min prior and 30, 60, and 90 min post-dose [83]. Previous studies indicated that OT can control zebrafish behavior in normal and stressful conditions [64]. A recent paper suggested the implication of this hormone in the development of the social behaviors in zebrafish when one OT receptor was knocked out. Moreover, it appears that the remaining receptors replaced the receptor’s activity by enhancing the formation of social responses, concomitantly leading to an elevated preference [55]. Also, the findings of Nunes et al. [84] revealed that the perturbation of OT receptors in the early stages of development can lead to a loss in dopaminergic neurons in zebrafish, and later, in the adult stage, they experienced an altered social response. Defective social decision making and decreased sociability were the main observations [84]. After zebrafish were kept in double-deionized water for one week in a generated stress environment, it was discovered that fish were presenting substantially more OT neurons than those from the control group [64].

Multiple lines of genetically modified animal models were used to study the relationship between OT and behavioral consequences. The first study, which assessed the OT alterations that resulted in social recognition memory deficits in a mouse model, also concluded the existence of different neural bases for social regulation [85]. Regarding the aggressive behavior of zebrafish, the present study recorded different effects depending on the exposure time. For instance, the intensity of aggression decreased significantly for the groups exposed only for fifteen minutes to OT in the first three days of exposure, but this was a short time effect. At the end of exposure, fish had demonstrated different trends; except for the fifteen group that received OT every day, the other groups maintained an elevated level in terms of time spent next to the stimulus. Evidence shows that early exposure to OT can contribute to an increase in mirror attacks per minute in zebrafish larvae treated with 50 µM OT for 24 and 48 h, respectively, at 100 µM OT for 24 h [56]. The current study highlights the importance of knowing the suitable duration of a treatment, and, in this context, it was indicated that OT exposure for just fifteen minutes for one week led to significant improvements in the swimming activity of zebrafish. In addition, aggression observed in zebrafish was time-dependent; continuous exposure to OT promoted a rise in this behavior when fish met the mirror in comparison to the initial behavior or even to the other groups. More evidence is required to establish the involvement of OT in zebrafish behavior when it comes to the type of administration, dosage, stage of development, and so on.

### 4.1. Limitations

While the current study sheds light on the potential of OT in modulating behavior in zebrafish, several limitations must be acknowledged. Firstly, the applicability of findings from zebrafish to humans is not straightforward due to inherent species-specific differences in neurobiology and behavior. Caution must be exercised in extrapolating these results to the context of human ASD. Additionally, our investigation was confined to a single dosage and varied exposure durations. Different dosages might produce distinct behavioral outcomes, necessitating further exploration. Moreover, this study’s focus on locomotor and aggressive behaviors leaves out other crucial behaviors related to ASD, particularly social interaction and anxiety-like behaviors. Lastly, the underlying neurobiological mechanisms by which OT influences these behaviors in zebrafish remain unexplored, presenting a gap in our understanding of its therapeutic potential in ASD.

### 4.2. Future Research

Future research directions should aim to address the limitations noted. Cross-species studies involving other animal models, and eventually human subjects, are imperative for validating the efficacy and safety of OT in ASD therapy. Investigating the impact of varying OT dosages and exposure durations on a broader spectrum of zebrafish behaviors could provide a more comprehensive understanding of its therapeutic window. The inclusion of assays that mimic ASD symptoms more closely, like social interaction and anxiety-related behaviors, would enhance the relevance of the findings to ASD. Integrating neurobiological approaches, such as brain imaging or neurochemical analyses, could unravel the mechanisms underlying OT’s behavioral effects. This might involve studying changes in neurotransmitter systems or gene expression patterns following OT treatment. Additionally, long-term studies on the effects of OT exposure on neurological development and behavior in zebrafish are needed. Such investigations would offer critical insights into the long-term implications of OT therapy in ASD.

In summary, while our findings contribute valuable knowledge about the influence of OT on zebrafish behavior, comprehensive research incorporating various behavioral aspects, dosages, neurobiological mechanisms, and long-term effects is essential to fully delineate OT’s potential as a therapeutic agent for ASD.

## 5. Conclusions

The focus of pharmacologic research on symptoms associated with autism spectrum disorder has been driven by the complex interaction of negative outcomes and the prevalence of aggressive behaviour in people with autism. Moreover, the use of oxytocin as a therapeutic intervention is often highlighted, but neurochemical and molecular analysis are needed. In the present work, oxytocin presented time-dependent effects that were seen in swimming activity and aggression level. Nevertheless, knowing the role of oxytocin in preclinical models is necessary to provide solid evidence of the long-term effects of treatment, dose optimization, time, and the mode of administration, and these should be monitored to discover the best intervention in autism with the help of oxytocin.

## Data Availability

No data availability for ethics reasons.
